# The impacts of urban–rural integrated medical insurance on the quality of labor supply for migrant workers in China

**DOI:** 10.1186/s12889-023-16839-6

**Published:** 2023-10-18

**Authors:** Xin Wang, Lele Li, Deshui Zhou

**Affiliations:** 1 School of Maritime Economics and Management, Gaoxin District, Dalian Maritime University, No.1 Linghai Road, Dalian, China; 2 School of Labor and Human Resources, Renmin University of China, No. 59 Zhongguancun Street, Beijing, China; 3 Institute of Finance and Public Management, Anhui University of Finance and Economics, No.1 Caoshan Road, Bengbu, China

**Keywords:** Urban and rural integrated medical insurance, Quality of labor supply, Migrant workers, Accessibility of public services

## Abstract

**Background:**

The transformation from the quantity of labor supply to the quality of labor supply is an important measure to improve the self-development of migrant workers.

**Method:**

Based on the 2018 China Floating Population Dynamic Monitoring Survey data, this paper uses the 2SLS model based on instrumental variable estimation to analyze the impact of urban and rural integrated medical insurance on the quality of migrant workers' labor supply.

**Results:**

The study found that: First, urban and rural integrated medical insurance can significantly improve the quality of labor supply for migrant workers. Even with different instrumental variables and the use of propensity score matching for counterfactual inferences, the findings remain robust. Second, the impact of urban–rural integrated medical insurance on the quality of labor supply for migrant workers has nonlinear characteristics. At the low quantile, the impact of urban–rural integrated medical insurance on the quality of labor supply for migrant workers showed a downward trend, but with the increase of the quantile, the impact of urban and rural integrated medical insurance continued to increase, showing a U-shaped trend.

**Conclusion:**

Urban–rural integrated medical insurance can not only directly reduce the labor time of migrant workers and ease the labor burden of migrant workers, but also indirectly improve the quality of labor supply for migrant workers through the intermediary role of promoting the availability of public services such as family contracted doctor services and health education.

## Introduction

In 2021, the National Bureau of Statistics released the 2020 China Economic Annual Report. The data showed that the total number of migrant workers in China was 285.6 million, of which 116.01 million local migrant workers, down 0.4%; and 169.59 million migrant workers, down 2.7%. Migrant workers are an important help to maintain China's rapid economic development, and have gradually become the main force of urban migrant workers in China. But for a long time, migrant workers have been a vulnerable group in society. The differences between urban and rural social security policies make the guaranteed treatment between urban and rural residents very different. The lack of social security level in rural areas undermines the fairness and justice of the medical insurance system. The mobility of migrant workers also makes it difficult to enjoy more complex procedures and lower levels of security at work sites because of different household registrations.

In China, the basic medical insurance system is an important tool to ensure the health of urban and rural residents and enhance the quality of workers. Its improvement and innovation greatly affect the operation and development of society. In order to break the problem of urban–rural dual division in the medical field, in 2016, the State Council issued the Opinions of the State Council on Integrating the Basic Medical Insurance System for Urban and Rural Residents, which clearly requires the integration of the two systems of urban residents' medical insurance and the new rural cooperatives into a unified urban and rural residents' medical insurance system, Level, facilitate the equalization of medical services.

In the context of the accelerating urbanization process, improving the quality of labor supply is the key to the reduction of the demographic dividend. Therefore, this article will focus on the impact of the implementation of urban and rural medical insurance coordination on the supply quality of migrant workers: compared with the previous one, has the implementation of the urban and rural medical insurance coordination system promoted the improvement of the labor quality of migrant workers? Has the unification of medical security rights and interests ameliorated the labor supply structure of migrant workers? What is the mechanism of urban and rural medical insurance affecting the quality of labor supply for migrant workers? Answering these questions can not only analyze the institutional effect of urban and rural medical insurance coordination from the theoretical level, but also provide empirical basis and reference for promoting the realization of the goal of high-quality labor supply for migrant workers.

## Literature review

Since rural residents possess a higher labor risk factor and a lower economic level, their health risks are higher than those of local urban residents, and their ability to resist diseases is relatively weak. From the perspective of medical treatment behavior, the medical security policy improves the residents' tendency to seek medical treatment through measures to compensate the insured residents' expenses, and expands the utilization rate of residents' medical services [1], thus improving the health level of residents [2]. However, at the same time, some studies believe that due to the small scope and low amount of reimbursement, it is inefficient and extremely limited in improving the health of rural residents [3], which can neither significantly affect the self-assesssment health of the rural registered population [4] or promote the medical treatment behavior of insured residents [5]. The inequality in the utilization level of medical services among urban and rural residents is actually an unequal opportunity [6, 7]. The new rural cooperative has a single financing channel and restricted subsidies. In the case of severe illness, the insufficient security for rural residents [8] cannot reduce the medical burden of rural residents, resulting in the failure of the risk-sharing mechanism. Moreover, because the primary medical and health system is still relatively weak, the health resources are insufficient, and there are few talents in the medical industry, the merger of township hospitals has reduced the provision of basic medical services, which are difficult to meet the needs of rural residents in rural areas.

The imperfection of the rural medical security system is the first dilemma faced by migrant workers, and the portability of the medical security system caused by the differences between urban and rural systems is the second dilemma encountered by migrant workers. Since the Reform and Opening up in 1978, a large number of farmers have been liberated from the land and poured into the city. Due to the division of the urban–rural dual system, the protection of the rights and interests of migrant workers has not been perfect [9]. Migrant workers need to participate in the new rural cooperatives in the place of household registration, and the insured place cannot change according to their construction site. Since the implementation of the new rural cooperative, China has always regarded the county as the basic unit for the overall planning and management of medical security funds. The fragmented operation of the overall planning fund makes the level of treatment between different overall planning regions different, and the guarantee is difficult to connect [10]. As a result of off-site reimbursement, migrant workers will face a higher starting point and a lower reimbursement ratio at the place of work.

The medical insurance system in the context of urban–rural division has led to the difficulties of migrant workers in medical services, hindered the free movement of migrant workers between rural and urban areas, and reduced the quality of migrant workers' labor. For a long time, the biggest obstacle for migrant workers to enter urban work has been long-term institutional barriers [11]. With the reform of the urban–rural dual system, the hindering effect of explicit household registration factors on migrant workers has gradually decreased, and the hidden hindering effect of social security factors related to them has begun to occupy an important position [12]. In the field of medical security, the new rural cooperative system with non-portable characteristics or discriminatory reimbursement policies is an institutional factor that significantly hinders the flow of rural labor [13]. Qin and Zheng [14] and other scholars have confirmed this with empirical research: the non-portable characteristics of the new rural cooperative system significantly reduces the rural labor force. The tendency to work hinders the free movement of rural labor to a certain extent. In addition, the pressure of lack of medical security may force migrant workers to carry out more preventive labor supply [15], thus prolonging the working hours of migrant workers and reducing their labor quality.

The difference in the level of medical insurance is easy to induce health loss in the rural registered population. The portability of medical insurance rights and interests also affects the allocation efficiency of migrant workers, which not only damages the fairness in the field of medical services, but also is not conducive to the sustainable construction and development of cities. The urban and rural medical insurance coordination system established in this context has promoted migrant workers to supply their own labor to cities. First of all, the urban–rural integrated medical system reduces the application procedures for reimbursement in different places under the original medical insurance system, increases the convenience of migrant workers to seek medical treatment in work places, declines the time loss brought by employment, reduces the phenomenon of rural registered population not seeking medical treatment when suffering from diseases [16], and increases the number of insured farmers The utilization rate of medical services and medical expenses of the village registered population have improved the self-assessment health and objective health of insured farmers [17]. Secondly, the urban–rural overall planning breaks the restrictions of urban and rural household registration and unifies the medical insurance treatment between urban and rural areas in the overall planning area [10], so that the medical insurance benefits that migrant workers can enjoy are not different from the change of their place of work, which improves the portability and accessibility of medical security, thus reducing the the loss of welfare benefits [18], and can play a positive role in guiding migrant workers' medical treatment, thus reducing the worries of migrant workers working in cities. At the same time, based on the theory of Social Integration, the overall planning of urban and rural medical insurance can promote the social integration of migrant workers at the mental health level by improving the accessibility of medical services, thus promoting the movement of migrant workers between urban and rural areas [6, 7]. In the specific research, Lu [19] also confirmed this point: participating in the overall planning of urban and rural medical insurance has a significant effect on the long-term migration willingness of the agricultural migrant population.

In summary, as an important tool to break the barriers between urban and rural areas, the social security system plays an important role in promoting the allocation of labor resources of migrant workers. The overall implementation of urban and rural medical insurance has promoted the free movement of migrant workers between urban and rural areas. Through sorting out the existing research, this paper finds that domestic scholars focus on analyzing the policy effect of the overall social security system, and mostly focus on the treatment differences between urban and rural residents or the choice of migration of the floating population. Its theoretical basis and empirical analysis are of reference significance to this article. However, few people conduct empirical analysis on the policy effect of a specific social security system, and research on the supply quality of migrant workers is even more rare. In view of this, this study will take solving the above shortcomings as the starting point and goal, using the 2018 China Mobile Population Dynamic Monitoring Survey Data (CMDS), analyze the impact of urban and rural medical insurance coordination on the supply quality of migrant workers through a 2SLS model based on tool variable estimation, and use the intermediary utility model to analyze its Impact mechanism.

## Methods

### Data source

The data of this article derives from the 2018 China Mobile Population Dynamic Monitoring Survey (CMDS) released by the National Health Commission. CMDS data is a special monitoring survey data for China's floating population. The target is a migrant population over 15 years old who have worked for more than one month in the place of relocation and are not registered in the place of residence. The survey scope of CMDS covers 31 provinces, autonomous regions and municipalities directly under the Central Government, with a size of about 160,000 samples, which is ample and representative. CMDS includes the income and expenditure of family members, employment, urban settlement, health, etc., which can provide better data support for the study of the socio-economic changes of China's floating population in the transition period. According to the research needs of this article, farmers with agricultural household registration (including agricultural transfer) and the age of 18–65 are selected as the research object. After excluding the relevant missing values and samples that do not meet the research requirements, the total number of samples used in this paper after controlling all variables is 79,981.

### Definition of variables

From Table 1, the explanation variable in this article is the urban–rural overall medical insurance. The urban–rural overall medical insurance is a combination of the new rural cooperative medical care and urban residents' medical insurance. In the corresponding questionnaire, "Do you have urban and rural residents' medical insurance", the "yes" is assigned to 1, and the no assignment is 0. And in order to compare the effect of urban and rural medical insurance, this article excludes the sample of insured urban employees. The average value of urban and rural overall medical insurance in the sample is 0.1255. The explanatory variable in this article is the quality of labor supply, which is measured by the hourly wage rate of migrant workers. The hourly wage level of migrant workers is a direct reflection of their unit productivity and an economic reflection of the labor income and employment quality of migrant workers. The specific calculation method is the ratio of the monthly employment income of migrant workers to the total number of working hours in four weeks. The hourly wage rate of migrant workers in the sample is 21.31 yuan.

**Table 1:** Descriptions of variables

Variable name	Variable-definition	Mean	Standard error
Quality of labor supply	Continuous variable of the hourly wage level of migrant workers	21.31	33.22
Overall urban and rural medical insurance	Urban and rural overall medical insurance = 1, no = 0	0.1255	0.3313
Sex	Male = 1 and female = 0	0.5085	0.4999
Marriage	Married = 1, no = 0	0.8071	0.3946
Age	Continuous variables for the age of the respondents	36.51	10.52
Educational level (primary school and below)			
Junior middle school	Junior high school = 1, no = 0	0.5003	0.5000
Senior middle school	High school = 1, no = 0	0.2065	0.4048
University and above	University and above = 1, no = 0	0.0840	0.2775
Family size	Count variable for the number of migrant worker families	3.2580	1.1978
County interval flow	County interval flow = 1, no = 0	0.1810	0.3851
Inter-provincial flow	Inter-provincial flow = 1, no = 0	0.4985	0.5000
Go out flow time	Cumulative years of migrant workers (years)	6.2260	6.1438
Self employment	Self-employment = 1, no = 0	0.4828	0.4997
Local long-term stay intention	Willing to live locally for a long time = 1, no = 0	0.8305	0.3752
Health status	Health status = 1, no = 0	0.8678	0.3387

In terms of control variables, the characteristics of gender, marriage, education, family size, mobility distance, mobility time, residence intention, self-employment and health status are controlled in turn, and the regional and unit characteristics of the interviewee are also controlled. Specifically, the gender variable is a two-category variable, the male assignment is 1, the female assignment is 0, and the average gender variable in the sample is 0.5085, indicating that the gender ratio of the sample interviewed is balanced. The marriage variable assigns a married spouse to 1 and an unmarried spouse to 0, with a mean value of 0.8071. Married samples account for a relatively large proportion. The age is a continuous variable, with an average value of 36.51 years old, indicating that the sample surveyed is relatively young overall. The education level variable shows that the sample ratio of junior high school, senior high school, college and above is 0.5003, 0.2065 and 0.0840 respectively, indicating that the proportion of migrant workers receiving college and above is low. The family size is the counting variable of the number of migrant workers, with an average value of 3. The family of migrant workers is miniaturized, that is, the separation between the left-behind father's family and the son's family. The average value of the flow of migrant workers in the county is 0.1810, and the proportion of inter-provincial flow is 0.4985, indicating that the scale of inter-provincial flow of migrant workers is relatively large. The average working time is 6.2 years, indicating that migrant workers are mainly migrant workers. The proportion of self-employment of migrant workers is 0.4828, indicating that flexible employment and individual self-employment are changing the traditional employment. The sample proportion of long-term willingness to settle in the local area is 0.8305, and the willingness of migrant workers to stay in the city is relatively high. The average health status is 0.8678, and migrant workers who go out self-evaluate their good physical health.

### Measurement method

Since the labor supply quality of migrant workers is a continuous variable, this paper first uses the OLS model to estimate and sets the following regression equations:

1 $${Labour\_sq}_{i}={\alpha }_{1}{X}_{i}+{\alpha }_{2}{Medical}_{i}+{\gamma }_{i}$$

In formula (1) ${Labour\_sq}_{i}$ indicates the quality of labor supply of the first migrant worker, ${Medical}_{i}$ indicating the participation of the first migrant worker in urban and rural overall medical insurance; ${X}_{i}$ represents a series of control variables. This article focuses on ${\beta }_{i}$. If it is significant, it shows that the overall medical insurance in urban and rural areas improves the quality of labor supply for migrant workers, and vice versa.

However, since the higher the quality of migrant workers' labor supply, it may also adversely affect their access to medical insurance rights and interests, it is estimated that OLS is not directly effective. In this regard, this article adopts the tool variable method to overcome it, and selects the urban and rural overall medical insurance participation rate of the interviewee's village except ‘myself’ as the tool variable. The overall medical insurance coverage rate at the village level is a higher-level variable for individuals. The participation behavior of others around them will affect the choice of individual behavior. The Peer Effect makes the tool variable have a significant correlation with the choice of individual behavior. However, the village and overall medical insurance participation rate are not directly related to the quality of individual labor supply, assuming that exogenous nature is established. In addition, in the robustness test, this paper introduces the interaction between the tool variable and the coverage ratio of occupational physicians at the residential level as the tool variable to increase the characteristics of exogenous disturbances and improve the robustness of tool variable estimation.

In addition, this paper also uses the intermediary effect model to estimate the mechanism of urban and rural overall medical insurance affecting the labor supply quality of migrant workers. And equations are set as follows:

2 $${Med}_{i}={\beta }_{1}{X}_{i}+{\beta }_{2}{Medical}_{i}+{\zeta }_{1}$$

3 $${Labour\_sq}_{i}={\gamma }_{1}{X}_{i}+{\gamma }_{2}{Medical}_{i}+{\gamma }_{3}{Med}_{i}+{\theta }_{1}$$

According to the intermediary effect test procedure, under the premise that the main effect in formula (1) is established, test the significance of formula (2) and formula (3). If both are significant, the intermediary effect is true; if at least one of the two is not significant, it is necessary to further test whether the indirect effect of the two is significant. If it is significant, the intermediate effect is therefore established.

## Results

### Benchmark regression

Table 2 is the benchmark return of the impact of urban and rural overall medical insurance on the quality of labor supply to migrant workers. After controlling a series of variables, the estimation results of the OLS and 2SLS models are reported respectively. The results show that the urban and rural overall medical insurance estimated based on the OLS model is significantly positive at the statistical level of 1%, which can significantly improve the labor supply quality of migrant workers by 4.11%. After considering the endogenous estimation and using the 2SLS model to control the endogenous, the statistical level of urban and rural overall medical insurance is still significantly positive at 1%, indicating that whether the OLS model is used or the 2SLS model estimated based on the tool variable method, urban and rural overall medical insurance can significantly ameliorate the labor supply quality of migrant workers. Research As a result, strong robustness is maintained. In addition, the F value of the tool variable based on 2SLS estimation is higher than the empirical value, so there is no weak tool variable problem.

**Table 2:** The impact of urban and rural integrated medical insurance on the quality of migrant workers' labor supply

Variables	OLS		2SLS	
	(1)	(2)	(3)	(4)
Overall urban and rural medical insurance	0.0482***	0.0411***	0.0585***	0.0538***
	(0.0068)	(0.0068)	(0.0095)	(0.0096)
Sex	0.2872***	0.2825***	0.2872***	0.2825***
	(0.0046)	(0.0046)	(0.0046)	(0.0046)
Marriage	0.0549***	0.0549***	0.0549***	0.0549***
	(0.0075)	(0.0074)	(0.0075)	(0.0074)
Age	0.0541***	0.0529***	0.0541***	0.0529***
	(0.0018)	(0.0018)	(0.0018)	(0.0018)
Age square	-0.0008***	-0.0008***	-0.0008***	-0.0008***
	(0.0000)	(0.0000)	(0.0000)	(0.0000)
Junior middle school	0.1456***	0.1469***	0.1455***	0.1468***
	(0.0065)	(0.0065)	(0.0065)	(0.0065)
Senior middle school	0.2840***	0.2873***	0.2837***	0.2871***
	(0.0080)	(0.0079)	(0.0080)	(0.0079)
University and above	0.4550***	0.4537***	0.4543***	0.4530***
	(0.0106)	(0.0106)	(0.0106)	(0.0106)
Family size	-0.0209***	-0.0221***	-0.0209***	-0.0221***
	(0.0024)	(0.0024)	(0.0024)	(0.0024)
County migration	-0.0515***	-0.0353***	-0.0515***	-0.0353***
	(0.0065)	(0.0065)	(0.0065)	(0.0065)
Inter-provincial migration	0.1182***	0.0657***	0.1184***	0.0660***
	(0.0051)	(0.0055)	(0.0051)	(0.0055)
Working time outside	-0.0029***	-0.0026***	-0.0029***	-0.0026***
	(0.0004)	(0.0004)	(0.0004)	(0.0004)
Self employment	-0.0341***	0.0478***	-0.0339***	0.0478***
	(0.0047)	(0.0061)	(0.0047)	(0.0061)
Will to stay in the city	0.0801***	0.0771***	0.0799***	0.0769***
	(0.0061)	(0.0060)	(0.0061)	(0.0060)
Health status	0.1068***	0.1027***	0.1068***	0.1026***
	(0.0077)	(0.0076)	(0.0077)	(0.0076)
Constant term	1.4144***	1.3924***	1.4133***	1.3914***
	(0.0321)	(0.0395)	(0.0321)	(0.0395)
Observed value	79,985	79,985	79,981	79,981
R square	0.1249	0.1386	0.1249	0.1385

***, ** and * are significant at the statistical level of 1%, 5% and 10%, respectively, with a standard error of robustness in parentheses

The results of the control variables in Table 2 are also noteworthy. Taking the 2SLS model as an example, the labor supply quality of male migrant workers is higher than that of women. Marriage variables show that the quality of labor supply for married migrant workers is higher than that of unmarried migrant workers. With the increase of age, the quality of labor supply of migrant workers is on the rise, but the square item of age is negative, indicating that with the further increase of age, the quality of labor supply will be reduced. Education variables show that the education of junior high school, high school and college and above is significantly positive, with coefficient values of 0.1468, 0.2871 and 0.4530 respectively, indicating that with the betterment of education, the quality of labor supply of migrant workers is on the rise. The larger the scale of migrant workers' families, the lower the quality of labor supply. Relocation within the county will reduce the quality of labor supply, but inter-provincial migration can significantly ameliorate the quality of labor supply for migrant workers. The longer people go out for work, the greater the quality of labor supply will be significantly reduced, which may be related to the enhance in the frequency of migrant workers. Compared with formal employment, the labor supply quality of self-employed migrant workers is low. In addition, the willingness and health status of long-term residence in the local area can significantly improve the quality of labor supply for migrant workers.

### Robustness test and counterfactual inference

From Table 3, this paper tests the robustness from the following three aspects: First, overcome the influence of outliers. In order to overcome possible outliers, this paper does 1% reduction before and after the sample. Secondly, eliminate the impact of development differences. In order to eliminate the influence of development differences between cities, this article excludes samples from Beijing, Shanghai, Shenzhen, Guangzhou and Tianjin. Then, upgrade the tool variable. In order to improve the exogenous nature of tool variables, this paper adopts the interaction between the rate of migrant workers in the village or community and the coordinated medical insurance in urban and rural areas as the tool variable. The introduction of interactive items can make the tool variables more exogenous. The above results show that no matter what kind of robustness test is used, the research results are significantly positive, indicating that the benchmark regression results of this article have strong robustness.

**Table 3:** Robustness test

Variables	Winsorization	Eliminate regional development differences	Upgrading IV
	(2)	(3)	(4)
Coordinated urban and rural medical insurance	0.0265***	0.0472***	0.0428***
	0.0086	0.0101	0.0144
Constant term	1.5420***	1.3292***	1.3666***
	0.0389	0.0424	0.0427
Observed value	76528	68,315	67,801
R-square	0.1407	0.1303	0.1435

*** , ** and * are statistically significant at 1%, 5% and 10%, respectively

In order to further overcome the impact of hybrid variables, this paper continues to use the tendency score matching method based on counterfactual inference for estimation. The equilibrium test results of the tendency score matching show that the absolute value of the standard deviation of most covariates after matching is reduced to less than 10%, which meets the matching conditions. The matching estimation results in Table 4 show that the ATT values based on the nearest neighbor matching, radius matching and kernel matching are significantly positive, and are basically consistent with the estimated values of the reference regression results. After overcoming the influence of hybrid variables, the research conclusion is still valid.

**Table 4:** Counterfactual inference based on propensity score matching

Matching method	ATT	Standard deviation	T statistics
Nearest neighbor matching (1:4)	0.0434***	0.0082	5.29
Radius Match (0.005)	0.0422***	0.0073	5.71
Core match (default value)	0.0443***	0.0073	6.02

***, **, and * are significant at the statistical levels of 1%, 5%, and 10%, respectively

### Quantity regression

Table 5 is the result of the decimal regression of the impact of urban and rural overall medical insurance on the quality of labor supply to migrant workers. Urban and rural overall medical insurance has a significant positive impact on different points. However, there are differences in the coefficient values of quantity regression on different segment points, which is difficult to observe in the OLS model and the 2SLS model. Specifically, at the 10th sub-point, urban and rural medical insurance can significantly improve the labor supply quality of migrant workers by 3.9%. At the 30th point, the overall medical insurance between urban and rural areas has significantly enhanced the quality of labor supply to migrant workers by 3.54%. At the 50th digit, the impact of urban and rural medical insurance has dropped to 3.05%. However, at a low of 70 points, the impact of urban and rural medical insurance on the quality of labor supply at a statistical level of 1% is on the rise. Further, at the 90th sub-location, the impact of urban and rural medical insurance on the labor supply quality of migrant workers increased significantly by 4.76%. According to these results, there are non-linear characteristics of the impact of urban and rural overall medical insurance on the labor supply quality of migrant workers. In the low situdrant, the impact of urban and rural overall medical insurance on the labor supply quality of migrant workers has shown a downward trend. However, with the improvement of the situd, the impact of urban and rural medical insurance increase, following U shape.

**Table 5:** Urban–rural pooling medical insurance and quality difference of migrant workers' labor supply: quantile regression

	Q10	Q30	Q50	Q70	Q90
Coordinated urban and rural medical insurance	0.0390***	0.0354***	0.0305***	0.0321***	0.0476***
	(0.0092)	(0.0085)	(0.0062)	(0.0082)	(0.0132)
Constant term	0.6661***	1.1940***	1.4455***	1.7112***	2.0468***
	(0.0749)	(0.0517)	(0.0435)	(0.0434)	(0.1172)
R-square	0.1139	0.0973	0.0875	0.0804	0.0729
Observed value	79,985	79,985	79,985	79,985	79,985

*** , **, and * are statistically significant at the 1%, 5%, and 10% levels, respectively, with robust standard error in parentheses

## Discussions

### The impact of urban and rural medical insurance on the working hours of migrant workers

The research in the previous article shows that urban and rural medical insurance can significantly ameliorate the labor supply quality of migrant workers. This part will continue to examine the direct impact of urban and rural medical insurance on the working hours of migrant workers. Working time is the guarantee for migrant workers to obtain economic benefits, but migrant workers generally face the problem of excessive working hours. On the one hand, excessively long working hours make migrant workers swamped with greater health losses, which is not conducive to continuous labor supply; on the other hand, obtaining labor income with longer working hours will reduce the production efficiency of migrant workers and reduce the sustainability of income acquisition. If the urban–rural overall medical insurance can not only improve the quality of the labor supply of migrant workers, but also reduce the working time of migrant workers, it means that the urban and rural overall medical insurance can significantly improve the labor supply structure of migrant workers, which is of significant economic significance. The test results are shown in Table 6. The coefficient values of urban and rural overall medical insurance in Model 1 and Model 2 are significantly negative, indicating that the urban and rural overall medical insurance significantly reduces the working hours of migrant workers, alleviates the labor burden of migrant workers, and thus improves the labor supply structure of migrant workers.

**Table 6:** The impact of pooling medical insurance in urban and rural areas on the working hours of migrant workers

Variables	Working hours	Working hours
Coordinated urban and rural medical insurance	-0.0167***	-0.0115**
	(0.0058)	(0.0058)
Constant term	4.0055***	4.0036***
	(0.0201)	(0.0255)
Miscellaneous Variables	control	control
Unit and regional characteristics	–	control
Observed value	80,392	80,392
R-square	0.0702	0.0947

*** , **, and * are statistically significant at the 1%, 5%, and 10% levels, respectively, with robust standard error in parentheses

### Analysis of the mechanism of urban and rural overall medical insurance affecting the quality of labor supply for migrant workers

Since urban and rural overall medical insurance can directly reduce the working hours of migrant workers and improve the quality of migrant workers' labor supply, this part will further discuss the mechanism of urban and rural overall medical insurance on the quality of migrant workers' labor supply. Migrant workers are a vulnerable group in the city, and their recognition of the city depends on the level of access to public services in the city. If urban public services can effectively benefit migrant workers, including contract services for family doctors’ inflow areas and relevant health education services, migrant workers will gain more sense of belonging and equal opportunities. Therefore, as an institutional arrangement to adjust the gap between urban and rural social security benefits, urban and rural overall medical insurance may indirectly affect the quality of labor supply by improving the availability of migrant workers' public services.

In view of this, this paper tests based on the intermediary effect model. In the selection of intermediary variables, the availability of public services for migrant workers is defined as whether to accept the contract services of the inflow family doctor and the level of health education. According to the design of the question "Do you enjoy the contract service of a family doctor locally" in the questionnaire, the answer will be "Yes" with a value of 1 and no value of 0. For the measurement of the level of health education, this article will include in the questionnaire, "Have you received local occupational disease prevention and control health education", "Have you received health education on infectious diseases", "Have received reproductive health education", "Have you received chronic disease prevention and control health education", "Have received mental health education?" For the question design of whether to accept health education for self-help in public emergencies and whether to accept health education in other aspects, first, reply to the above variables as "yes" and assign 1 to "no" to 0 in turn. Then add up each virtual variable to build a continuous variable of health education level 1 to 7. The higher the score after the sum, the higher the level of health education that migrant workers receive.

The results of the intermediary effect test are shown in Table 7. Column (1) and column (3) respectively test the impact of urban and rural overall medical insurance on intermediary variables, that is, the impact on family contracted doctor services and health education. Column (2) and column (4) respectively include the above variables in the regression equation of the impact of urban and rural overall medical insurance on the quality of labor supply to test whether the intermediary effect exists. The results show that the values of column (1) and column (3) are significantly positive, indicating that the overall medical insurance in urban and rural areas can significantly improve the probability of migrant workers receiving the contract services of family doctors in the place of relocation, as well as the probability of receiving health education. In columns (2) and (4), family doctor signing services and health education are included in the regression equation, and the results show that the overall medical insurance in urban and rural areas is still significantly positive. And after adding intermediary variables to column (2) and (4), the influence coefficient of urban and rural overall medical insurance has decreased to a certain extent compared with the benchmark regression (0.0538), indicating that the intermediary variable dilutes the positive impact of urban and rural overall medical insurance on the labor supply quality of migrant workers to a certain extent. That is, urban and rural overall medical insurance can indirectly improve the quality of labor supply by increasing the probability of migrant workers receiving contract services from family doctors in the place of moving to, as well as the probability of receiving health education.

**Table 7:** Test results of mediating effect

Variables	(1)	(2)	(3)	(4)
	Family contracted physician services	Quality of labor supply	Health education	Quality of labor supply
Medical insurance for urban and rural residents	0.0191***	0.0420***	0.3326***	0.0515***
	(0.0070)	(0.0100)	(0.0289)	(0.0096)
Family doctor contract services		0.0580***		
		(0.0055)		
Health education				0.0070***
				(0.0013)
Constant term	0.0567**	1.4542***	1.0974***	1.3836***
	(0.0288)	(0.0417)	(0.1171)	(0.0396)
Observed value	72,776	72,129	80,705	79,981
R square	0.0116	0.1463	0.0561	0.1388

***, ** and * are significant at the statistical level of 1%, 5% and 10%, respectively, with a standard error of robustness in parentheses

## Conclusions and recommendations

The transformation from the quantity of labor supply to the quality of labor supply is an important measure to ameliorate the self-development of migrant workers. This paper uses the dynamic monitoring survey data of China's floating population in 2018 and analyzes the impact of urban and rural overall medical insurance on the quality of labor supply for migrant workers based on the 2SLS model estimated by tool variables. The study found that: First, the overall medical insurance in urban and rural areas can significantly improve the quality of labor supply for migrant workers. Even if the interpreted variables are defined in different ways, the tool variables are upgraded, and the tendency score matching method is used for counterfactual inference, the research conclusion is still solid. Second, the impact of urban and rural overall medical insurance on the labor supply quality of migrant workers has nonlinear characteristics. Based on the estimation of the decimal model, it is found that at the low quinox, the impact of urban and rural overall medical insurance on the labor supply quality of migrant workers has shown a downward trend. However, with the betterment of the quinox, the impact of urban and rural overall medical insurance continues to increase, showing a U-shaped trend. Third, the coordinated medical insurance in urban and rural areas can not only directly reduce the working hours of migrant workers and alleviate the labor burden of migrant workers, but also indirectly improve the labor supply quality of migrant workers by promoting the intermediary role of family contract doctor services, health education and other public services.

The study of this article has important policy implications: First, improving the level of overall medical insurance in urban and rural areas is an inherent requirement to promote the health rights and interests of migrant workers. Urban health rights and interests should be strengthened to benefit migrant workers and provide social security and shelter for migrant workers, so as to facilitate the quality of migrant workers' labor supply. Second, the transformation of the quantity of labor supply to the quality of labor supply of migrant workers should be promoted. This paper also finds that when the quality of labor supply is at a high level, the impact of urban and rural overall medical insurance has increased significantly, indicating that the quality of labor supply is related to the high-level preservation of urban and rural overall medical insurance, so as to broaden Providing labor protection for migrant workers is an effective way to promote the reach of the health rights and interests of migrant workers and the quality of labor supply. Third, accelerate the equalization of basic public services. Improve the signing services of migrant workers' family doctors, strengthen the training of migrant workers' health education, protect the labor rights and interests of migrant workers, and provide policy guarantees for improving the quality of migrant workers' labor supply.

## Data Availability

The data is not publicly available. The datasets used and/or analysed during the current study available from the corresponding author on reasonable request.
